# Pericardial effusion with cardiac tamponade caused by a central venous catheter in a very low birth weight infant

**DOI:** 10.11604/pamj.2016.25.13.8731

**Published:** 2016-09-20

**Authors:** Fatma-Zohra Chioukh, Karim Ben Ameur, Hayet Ben Hmida, Kamel Monastiri

**Affiliations:** 1 Department of Intensive Care and Neonatal Medicine, Teaching Hospital of Monastir, Tunisia

**Keywords:** Newborn, umbilical central venous catheterization, pericardial effusion, cardiac tamponade, pericardiocentesis

## Abstract

With more and more extreme premature and very low-birth weight babies being resuscitated, umbilical central venous catheterisation is now being used more frequently in neonatal intensive care. One of the life-threatening complications is pericardial effusion and cardiac tamponade; however, it is potentially reversible when it is caught in time. The authors present a case of cardiac tamponade following umbilical venous catheterisation in a neonate. The patient was diagnosed at the appropriate time by echocardiography and urgent pericardiocentesis proved lifesaving.

## Introduction

Central venous catheters (CVC) are frequently used in neonatal intensive care especially in premature babies. They are relatively easy to insert and provide secure venous access for a longer duration. However, they can cause lots of complications such as pericardial effusion with cardiac tamponade (PE/CT) [1]. We report one case of cardiac tamponade that was diagnosed using a transthoracic echocardiography, followed by successful urgent needle pericardiocentesis.

## Patient and observation

A preterm baby girl was born at 27 weeks gestation to a 31-year-old mother by cesarean section. Birth weight was 970 grams and Apgar scores were 6 at one minute and 7 at five minutes. She was admitted to neonatal intensive care unit for respiratory distress syndrome requiring nasal ventilation. On the second day of hospital stay, the infant had venous umbilical catheter inserted (epicutaneo-Haumont-kit 24G-2Fr-Vygon). X-ray abdomen and chest was done to confirm the position of the CVC.

**Figure 1:**
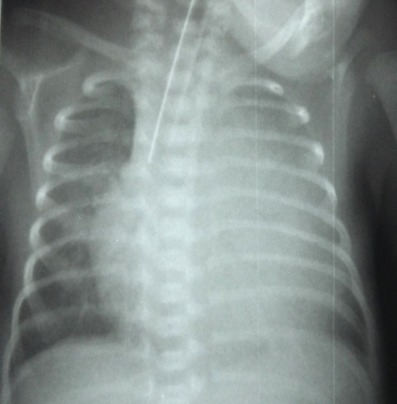
Chest x-ray showing increase of the cardiac area

**Figure 2:**
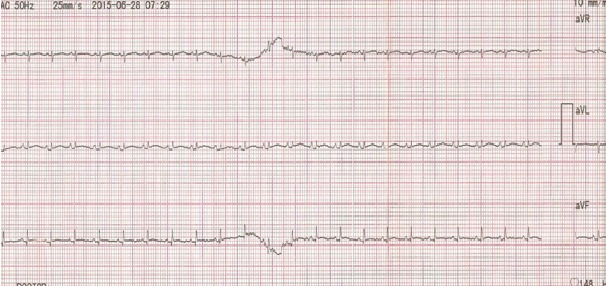
Electrocardiogram showing microvoltage

**Figure 3:**
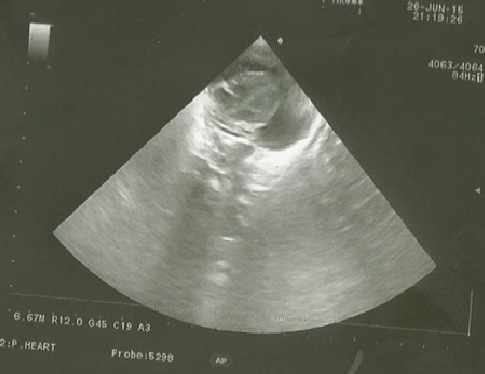
Four chamber echocardiography view showing a significant pericardial effusion

**Figure 4:**
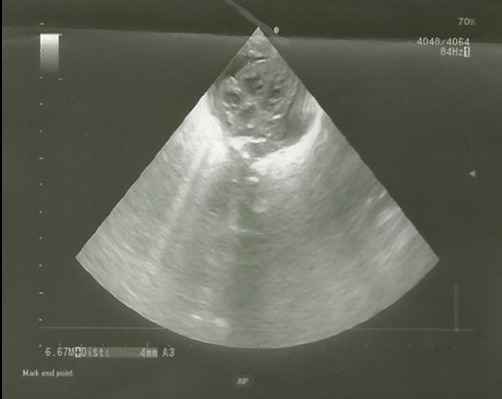
Reduction of pericardial effusion on echocardiogram immediately after pericardiocentesis

After 3 days of catheter implantation, the patient’s cardiorespiratory condition worsened, and there was an increase of the cardiac area on chest radiography (Figure 1). The baby was intubated immediately and one boluse of normal saline for low blood pressure was required. Electrocardiogram showed a low voltage (Figure 2). Transthoracic echocardiogram showed large pericardial effusion (Figure 3). All fluids through the umbilical catheter were discontinued and a peripheral intravenous line was started. Urgent subxiphoid pericardiocentesis with a scalp vein was performed bedside under echographic guidance. Twenty ml of clear liquid was drained. The fluid was sent for culture which was negative. Proteins and lactate dehydrogenase were normal. Microscopy showed occasional red and white blood cells. An echocardiogram was done after the procedure revealed partially resolution of the pericardial effusion (Figure 4). The newborn was extubated on the 10^th^ post pericardiocentesis day.

## Discussion

Diagnosis of pericardial effusion in our preterm baby was primarily suspected by an unexplained cardiorespiratory instability and an increased cardiac silhouette. Patients with such complication are often presented with sudden cardiac arrest [2]. Pericardial effusion development usually occurs on the 3^rd^ or 4th day after catheterization as in our patient [3].

The 2001 Manchester Report from the United Kingdom recommended that all central venous lines inserted specifically for parenteral nutrition in neonates should be placed outside the cardiac chambers [4]. In 2002, the FDA (U.S. Food and Drug Administration) recommended that the catheter tip should not be placed in the right atrium, and that the neonate’s movements should be minimized in order to keep the catheter from migrating into the RA [5]. Even after these recommendations were released, neonatal PE/CT cases related to CVC use continued to be reported [6]. Among the reported cases were some with appropriate CVC tip positioning, as in our patient [7–9].

Prematurity is an additional risk factor, as the methods used to estimate the insertion length are not satisfactory to guide to the exact position in premature infants [10]. Furthermore, the repositioning of CVC due to weight loss and consequently decreased abdominal girth may result in complications related to catheter migration in very low-birth weight newborns [11]. An autopsy series of neonatal PE/CT related to CVC described right atrial histopathologic findings in all cases and included a case with appropriate CVC tip positioning [2]. Right atria in PE/CT demonstrated marked interstitial edema and dilated fine vascular channels. Endocardial injury with permeation of hyperosmotic total parenteral nutrition fluid into the interstitium and egress into the pericardial sac is hypothesized as the etiology of PE.

As in our case, expedient recognition of PE using echocardiographic ultrasound followed by a pericardiocentesis may avoid sudden unexpected deaths in neonates [12].

## Conclusion

Cardiac tamponade as a complication caused by the umbilical vein catheterization in newborns is rare, but serious and can occur even in cases in which the catheter is properly positioned. The neonatologist should be aware of catheter tip placement and consider PE/CT in a neonate with sudden cardiac decompensation. Immediate pericardiocentesis may remove the PE and avoid sudden death.
